# Meningioma Grading beyond Histopathology: Relevance of Epigenetic and Genetic Features to Predict Clinical Outcome

**DOI:** 10.3390/cancers15112945

**Published:** 2023-05-27

**Authors:** Elena Marastoni, Valeria Barresi

**Affiliations:** Department of Diagnostics and Public Health, University of Verona, 37134 Verona, Italy; marastoni.elena@gmail.com

**Keywords:** meningioma, methylome, recurrence, grading, ACADL, MCM2, *CDKN2A*, *pTERT*, proteomic, transcriptomic

## Abstract

**Simple Summary:**

Meningiomas are common tumors of the central nervous system. The grading system established by the World Health Organization has recently included *pTERT* mutations and *CDKN2A/B* homozygous deletions as criteria for grade 3, owing to their close association with increased recurrence risk. However, these alterations identify only a part of meningiomas that are devoid of histopathological malignancy and are prone to recurrence. This review summarizes the most recent knowledge on the molecular landscape of meningiomas, according to which these tumors can be classified into three main groups, showing distinct clinical outcomes and epigenetic, genetic, transcriptomic, and proteomic features. There is some evidence that these groups can be distinguished in routine practice using specific immunostaining and may likely be treated with different and targeted approaches.

**Abstract:**

Meningiomas are common tumors of the central nervous system. The grading system established by the World Health Organization (WHO) has recently included *pTERT* mutations and *CDKN2A/B* homozygous deletions as criteria for grade 3, owing to their association with increased recurrence risk. However, these alterations identify only a portion of meningiomas that are devoid of histopathological malignancy and are prone to recurrence. Over the last few years, the integration of epigenetic, genetic, transcriptomic, and proteomic profiling has led to the identification of three main groups of meningiomas with distinct clinical outcomes and peculiar genetic features. Meningiomas in the first group have the best prognosis, are distinguished by the lack of *NF2* alterations and chromosomal instability, and may be responsive to cytotoxic drugs. Meningiomas in the second group have an intermediate prognosis and are characterized by *NF2* alterations, mild chromosomal instability, and enrichment in immune cells. Meningiomas in the third group had the worst prognosis, displayed *NF2* alterations coupled with high chromosomal instability, and were resistant to cytotoxic treatment. Classification into these three groups predicts the recurrence risk of meningiomas more accurately than WHO grading and could be applicable in routine practice, owing to the possibility of distinguishing the different groups by specific immunostaining.

## 1. Introduction

Meningiomas account for approximately 40% of central nervous system (CNS) tumors [1]. Although they are mostly benign and indolent [1], a percentage recurs or even shows malignant progression and poor outcomes [2]. The extent of surgical resection [3] and the three-tiered World Health Organization (WHO) histopathological grading are considered major prognostic factors of recurrence and overall survival [4] and guide the post-surgical treatment of patients with meningiomas [2]. According to the current guidelines, waiting and observation are indicated for patients with grade 1 meningioma, whereas radiotherapy is indicated for patients with grade 3 meningioma [2]. Patients with subtotal or partial resection of grade 2 meningiomas are invariably treated with adjuvant radiotherapy, whereas the post-surgical treatment of grade 2 meningiomas that undergo gross total resection remains controversial, and either radiotherapy or observation is suggested [2]. Predicting the recurrence risk of grade 1 and 2 meningiomas after complete surgical resection is currently a major issue. For this reason, over the last 10 years, research has focused on the identification of other predictors of the clinical outcome of meningiomas and mainly on genetic or epigenetic factors that could better reflect the biological aggressiveness of these tumors than their histopathological features.

This review aimed to summarize the main changes in the criteria for meningioma grading brought about by the fifth edition of the WHO Classification of CNS Tumors, the limits of the current CNS WHO grading system, and its possible future evolution with the inclusion of other significant prognostic molecular features. 

## 2. Meningioma Grading in the Fifth Edition of WHO Classification (WHO 2021)

In the updated fourth edition of the WHO Classification (WHO 2016), meningioma grading was based only on histopathological features, and each histotype was assigned its own grade [5] (Table 1).

Among the meningiomas in the Central Brain Tumor Registry of the United States, 80.1% were grade 1, 18.3% were grade 2, and 1.5% were grade 3 [1]. The recurrence risk was 7–25% for grade 1, 29–52% for grade 2, and 50–94% for grade 3 meningiomas [5], in accordance with the strong prognostic value of the WHO grading system.

To improve its prognostic significance, the WHO grading of meningiomas was modified in the fifth edition of the Classification (WHO 2021) of CNS Tumors, with the inclusion of molecular features associated with a worse outcome as criteria for grade 3. In addition, in contrast to WHO 2016, meningioma is now considered a single tumor type with different histological subtypes and grades [4] (Table 1). This means that, with the exception of chordoid and clear cell subtypes, which are designated as grade 2 in all cases, all other subtypes are graded according to specific histopathological and/or molecular parameters [6,7,8]. In detail, meningiomas are classified as grade 2 in the presence of: (i) at least four mitoses in ten consecutive high-power fields (HPF) of 0.16 mm^2^; and/or (ii) brain invasion; and/or (iii) at least three parameters among spontaneous necrosis, patternless growth (sheeting), macronucleoli, hypercellularity, and small cells with a high nuclear/cytoplasmic ratio [4]. Meningiomas are considered grade 3 when they have 20 or more mitoses in 10 HPF of 0.16 mm^2^, and/or frank histological anaplasia with a morphology resembling a carcinoma, melanoma, or sarcoma, and/or *TERT* promoter (*pTERT*) mutation, and/or *CDKN2A*/*B* homozygous deletion (HoDe) [4].

## 3. Worth and Limits of WHO 2021 Grading

The WHO 2021 Classification has the merit of introducing genetic alterations into the meningioma grading system for the first time. This allows the identification of at least a percentage of tumors harboring a high risk of progression or recurrence despite the lack of histopathological features of malignancy. Indeed, different studies have demonstrated that *pTERT* mutations are strongly associated with the recurrence and/or progression of meningiomas to a higher grade or reduced overall survival [9,10,11,12,13,14,15,16]. In addition, *CDKN2A/B* HoDe was introduced as a criterion for upgrading meningiomas to grade 3, owing to its significant association with recurrence and shorter progression time [17,18,19,20,21]. 

However, both these genetic alterations are infrequent in meningiomas histologically classified as grade 1 or 2. Indeed, in a meta-analysis of 677 patients, only 8/169 (4.7%) grade 1 and 29/365 (7.9%) grade 2 meningiomas (classified according to WHO 2016) had *pTERT* mutations [11]. *CDKN2A/B* HoDe was absent in 238 grade 1 cases, present in only 7/213 (3.2%) grade 2 meningiomas [10], and found in only 30/1358 (2.2%) grade 1 and 2 cases in another series [22].

In addition, *pTERT* mutations and *CDKN2A*/*B* HoDe identify only some meningiomas that are devoid of histological malignancy but are prone to recurrence. Indeed, only 2/32 (6.2%) grade 1 and 5/39 (12.8%) grade 2 recurring meningiomas (classified according to WHO 2016) had *pTERT* mutations in a study of 252 patients [10]. In addition, *CDKN2A/B* alterations were found in only 13.1% of recurring tumors in an analysis of 583 meningiomas [18] and in only 1 of 12 recurring atypical meningiomas (classified according to WHO 2016) by our group [19]. 

Therefore, although the inclusion of these molecular features in meningioma grading is helpful in identifying histologically non-malignant tumors that harbor a higher recurrence risk, the current WHO classification mostly leaves the issue of predicting the recurrence risk of grade 1 and 2 meningiomas, which is essential for establishing appropriate treatment after surgery. Notably, a recent analysis of 776 meningiomas showed that even heterozygous deletion of *CDKN2A/B* was associated with a shorter time to recurrence [18], which was confirmed in another series of 1506 cases [19]. Apart from the inclusion of molecular features among the criteria, the current WHO grading of meningiomas has remained substantially unchanged compared with that of WHO 2016. Brain invasion is still considered a sufficient criterion for classifying meningiomas as grade 2 [4], although several studies have suggested that meningiomas classified as grade 2 owing to the presence of brain invasion, but lacking a high mitotic index, have a recurrence risk overlapping with that of grade 1 meningiomas [23,24,25].

## 4. DNA Methylation Profiling for Meningioma Grading

DNA methylation profiling has been used for the classification and identification of new types of CNS tumors. Indeed, different tumors have distinct DNA methylation profiles depending on their cell of origin and the molecular alterations acquired during progression [26].

To identify predictors of the clinical outcome of meningiomas that are more accurate than the WHO grading system, meningiomas have been profiled for DNA methylation in several studies.

In 2012, Kishida et al. first demonstrated the potential prognostic significance of DNA methylation profiles in meningiomas, reporting a higher number of methylated genes in recurrent tumors than in nonrecurrent tumors [27]. Thereafter, in 2017, Olar et al., in a training cohort of 89 tumors and a validation set of 51 tumors, demonstrated that meningiomas clustered into two subgroups characterized by distinct methylation patterns and different prognoses [28]. Meningiomas in the prognostically unfavorable (MM-UNFAV) subgroup harbor a higher number of methylated genes and chromosomal copy number variations (including recurrent losses of 1p, 6q, 14q, and 18q, and gain of 1q), are mostly high-grade, and have shorter recurrence-free survival than meningiomas in the prognostically favorable (MM-FAV) subgroup [28] (Table 2).

Finally, based on the DNA methylation profiling of 497 meningiomas, Sahm et al. developed a methylation-based classification that predicted tumor recurrence with a higher power than the WHO 2016 grading [29]. In their analysis, meningiomas were clustered into two main epigenetic groups, A and B, which featured distinct prognoses. Group A included three methylation classes (MC) with a benign clinical course (MC ben-1, MC ben-2, and MC ben-3) and one MC with an intermediate prognosis (MC int-A), whereas group B included one MC with an intermediate prognosis (MC int-B) and one with malignant behavior (MC mal) [29]. After integrating epigenetic and clinical data, meningiomas were classified into three main combined methylation classes: (1) benign, including three methylation classes clinically characterized by indolent behavior (MC ben-1, MC ben-2, and MC ben-3); (2) intermediate, including two methylation classes (MC int-A and MC int-B) with intermediate outcomes; and (3) malignant, including meningiomas in group B with aggressive clinical behavior (MC mal) [29] (Table 2). Combined methylation classes partially overlap with WHO grades; indeed, the majority of grade 1 meningiomas cluster in MC benign, whereas most grade 3 meningiomas fall into MC malignant [29]. However, clinically aggressive grade 1 meningiomas cluster in MC intermediate, and indolent atypical (WHO grade 2) meningiomas are classified as MC benign, thus demonstrating the superiority of methylation-based classification in predicting recurrence risk compared to the WHO grade [29]. 

Notably, these three combined methylation classes feature distinctive genetic alterations [29,31]. Indeed, mutations in *SMO*, *KLF4*, *AKT1*, *PIK3CA*, and *TRAF7* are exclusive to MC benign, whereas chromosomal copy number aberrations (including 1p, 6q, 10q, 14q, and 18q heterozygous deletions), as well as *CDKN2A/B* HoDe and *pTERT* mutations, are more frequent in MC intermediate and malignant [29,31].

Despite its higher prognostic relevance compared to WHO grading, methylation-based classification of meningiomas may not be applicable in routine practice on a large scale, owing to its high cost and the need for sophisticated instruments. Notably, a study of 87 meningiomas showed that an epigenetic modification, consisting of the loss of trimethylation of lysine 27 of histone H3 (H3 K27me3), is significantly associated with methylation group B and correlates with a higher recurrence risk [30]. The prognostic role of H3 K27me3 loss in meningiomas of grades 1 and 2 has been confirmed in other studies [32,33], and it likely predicts the recurrence risk after stereotaxic radiosurgery [34].

Although H3 K27me3 loss can be easily demonstrated using immunohistochemistry and a specific antibody, its routine assessment may be hampered by heterogeneity and difficulty in interpreting immunostaining results [35].

## 5. Integrated Molecular–Morphological Grading

The close correlation among methylation class, WHO grade, and genetic alterations has led to the search for new grading systems that integrate this information.

**Figure 1:**
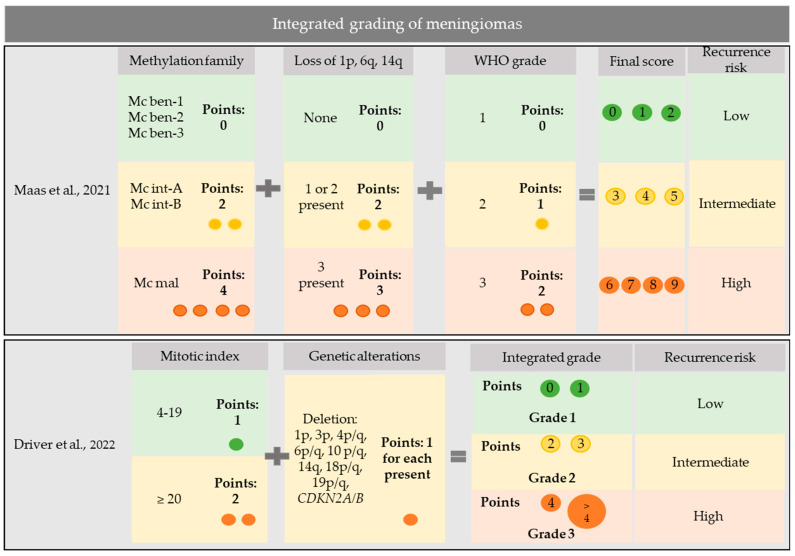
Integrated grading systems for meningiomas. Maas et al., 2021 [31]. Driver et al., 2022 [36].

In a discovery cohort of 514 meningiomas and a validation set of 471, which were profiled for DNA methylation, gene mutations, and chromosomal copy number aberrations, Maas et al. developed an integrated three-tiered grading system for meningiomas [31]. To grade meningiomas, they considered all variables showing the highest prognostic significance for recurrence risk: WHO grade, combined methylation class, and loss of chromosomes 1p, 6q, and 14q [31]. Meningiomas were graded by assigning 0–2 points to WHO grading, 0–4 points to combined methylation class, and 0–3 points to chromosomal loss, thus obtaining a final score ranging from 0 to 9 (score 0–2: low risk; score 3–5: intermediate risk; and score 6–9: high risk) [31] (Figure 1).

This integrated score predicted the recurrence risk of meningiomas with higher accuracy than the WHO grade, chromosomal copy number aberrations, or combined methylation classes [31]. Notably, some meningioma subtypes fell only in one risk category; in particular, meningiomas of angiomatous (14/14 cases), psammomatous (23/23 cases), and secretory (24/24 cases) subtypes had an integrated score consistent with a low risk in all cases, whereas meningiomas of the clear cell subtype (43/43 cases) invariably displayed an integrated score corresponding to an intermediate risk [31]. Therefore, molecular prognostic stratification can be amended for these subtypes, and reserved for other meningioma subtypes. In accordance with previous findings [29], mutations in *SMO*, *KLF4*, *AKT1*, *PIK3CA*, and *TRAF7* were exclusive to the benign methylation class, suggesting that their presence could be used as a surrogate for the methylation profile to identify benign meningiomas [31].

The integrated grading proposed by Mass et al. was based on the 2016 WHO grading system [31]. A recent study published by the same research group demonstrated that using the 2021 WHO grade for the integrated score does not produce any substantial changes; therefore, according to this result, there is no need for additional testing for *pTERT* and/or homozygous losses of *CDKN2A/B* when defining the integrated score in risk prediction for meningioma patients [37].

Driver et al. proposed an alternative integrated molecular grading system based on the mitotic count, *CDKN2A/B* HoDe, and chromosomal copy number aberrations [36]. In detail, they assigned one point to each genetic alteration among 1p, 3p, 4p/q, 6p/q, 10p/q, 14q, 18p/q, and 19p/q deletions, and *CDKN2A/B* homozygous or heterozygous deletions, as well as one point to a mitotic index of 4–19 mitoses/1.6 mm^2^ or two points to a mitotic index of ≥20 mitoses/1.6 mm^2^ [36]. They then classified meningiomas with 0–1 point as integrated grade 1, meningiomas with 2–3 points as integrated grade 2, and meningiomas with ≥4 points as integrated grade 3 [36] (Figure 1). In a discovery cohort of 527 meningiomas and a validation set of 172 meningiomas, this integrated molecular grading was superior to the WHO grading for predicting recurrence risk [36]. In addition, it nicely stratified WHO grade 2 meningiomas, which resolved in integrated grade 1 in one-third and in integrated grade 3 in another third [36], with potential application in deciding adjuvant treatments.

## 6. Molecular Classification of Meningiomas

In a transcriptomic study of 146 meningiomas of different WHO grades, Patel et al. identified three main molecular types (A, B, and C) that had distinct genetic features and predicted recurrence risk with higher accuracy than the WHO grade [38]. Type A tumors mostly include WHO grade 1 tumors, which lack *NF2* mutations and chromosomal copy number aberrations and have *TRAF7*, *KLF4*, *AKT1,* and a low recurrence rate. Type B meningiomas feature *NF2* mutations and 22q loss, and have a recurrence rate similar to that of type A. Finally, type C meningiomas have *NF2* mutations associated with high genomic instability, consisting of frequent chromosomal losses (among which 22q and 1p losses are the most frequent), and feature the worst prognosis [38]. Type B and C meningiomas were additionally distinguished by the loss of repressor function of PRC2 in the former and of the DREAM complex in the latter [38] (Table 3).

In 2021, Nassiri et al. combined findings obtained with DNA somatic copy number aberrations, DNA somatic point mutations, DNA methylation, and messenger RNA abundance in 201 meningiomas of different WHO grades to identify four consensus molecular groups showing distinctive genetic alterations and proteomes [39], and named them immunogenic (MG1), benign NF2-wildtype (MG2), hypermetabolic (MG3), and proliferative (MG4) [39]. Meningiomas in MG1 had the longest recurrence-free survival, invariably featured *NF2* biallelic inactivation due to co-occurring *NF2* mutation and 22q loss, lacked other chromosomal alterations, and featured greater immune infiltration and enrichment of pathways involved in immune regulation and signaling [39]. Meningiomas in MG2 were invariably *NF2* wild type and included two main subgroups: one with *KLF4*, *TRAF7*, or *AKT1* mutations, and another displaying the polysomy of chromosomes 5, 12, 13, and 20 [39]. Meningiomas in MG3 and MG4 had the worst prognosis, were enriched in mutations in chromatin remodeling and tumor suppressor genes, and featured high aneuploidy with frequent losses in 1p, 6q, 14q, 18q, and 22q [39]. 

Notably, stratification by the molecular group had higher accuracy in predicting recurrence risk than the WHO grade or methylation-based classification [39]. Although this classification is derived from sophisticated analyses, it can be applied in routine practice because molecular groups are distinguished by the expression of different proteins (MG1 by S100A, MG2 by SCGN, MG3 by ACADL, and MG4 by MCM2) that can be detected using immunohistochemistry and specific antibodies [39]. In a recent study of 55 primary atypical meningiomas, we showed that ACADL and MCM2 immunostaining, used as surrogates for MG3 and MG4, predicted shorter recurrence-free survival and was associated with a higher mitotic index and 1p and 18q losses [41]. 

A different study, published in 2022 by Choudhury et al. [40], integrated the DNA methylation profiling of 565 meningiomas and genetic, transcriptomic, biochemical, proteomic, and single-cell analyses and achieved similar findings to those of Nassiri et al. [29]. They showed that meningiomas can be stratified into three methylation groups with distinct clinical outcomes, biological drivers, and possible therapeutic vulnerabilities [40]. The NF2/merlin intact group likely corresponds to the MG2 (NF2 wild-type) group described by Nassiri et al.; it features the best prognosis and may be responsive to cytotoxic therapies, owing to the preserved apoptotic function of merlin [40]. The immune-enriched group, overlapping MG1 (immunogenic), has an intermediate outcome, displays inactivation of *NF2* by 22q loss, and is distinguished by enrichment of infiltrating immune cells and lymphatic vessels [40]. Finally, the hypermitotic group, corresponding to MG3 and MG4, has the worst prognosis and is distinguished by high aneuploidy with frequent chromosomal losses, *CDKN2A/B* HoDe, hypermethylation, and resistance to cytotoxic therapies [40]. In a recent study, Choudhury et al. confirmed that their hypermitotic group can be further divided into two subgroups: proliferative, enriched in the expression of genes driving cell proliferation and corresponding to MG4, and hypermetabolic, enriched in the expression of genes driving macrometabolism and corresponding to MG4 [40]. 

The studies mentioned above demonstrate that meningiomas can be stratified into biologically and clinically distinct groups according to their DNA methylation and transcriptomic profiles. In 2022, Bayley et al. demonstrated that the findings achieved using these two approaches were highly concordant [42].

They performed DNA methylation profiling of 110 meningiomas (WHO grades 1 and 2), blinded to the results of transcriptional profiling (which classified meningiomas as described in the previous paragraph), and obtained three clusters of increasing malignancy: (1) Meth1, showing a balanced methylation pattern; (2) Meth2, featuring hypomethylation; and (3) Meth3, distinguished by hypermethylation in promoter CpG islands [42]. They additionally classified meningiomas according to their copy number variations as having no loss, 22q loss, or 1p/22q loss, with the latter displaying the worst prognosis [42]. A comparison of the three classification approaches showed an agreement rate of 75%. In other words, 75% of meningiomas were considered in the same risk category across all three classifications [32]. Finally, meningiomas were distinguished based on *NF2* status and chromosomal instability (CIN) as “NF2-intact”, “NF2-deficient, low CIN (one or two chromosomal deletions)”, and “NF2-deficient, high CIN (more than two chromosomal deletions)” [32]. The four classifications had a concordance rate of 70% [42].

This study suggests the presence of three distinct groups of meningiomas with unique clinical features:

– meningioma group A (MenGA), mainly consisting of WHO grade 1 meningiomas, with female preponderance, NF2-intact, a low frequency of necrosis, low proliferation, and an indolent clinical course;
– MenGB, mostly formed of WHO grade 1 meningiomas, with female preponderance, NF2-deficiency, a low frequency of necrosis, low proliferation, and an indolent clinical course;
– MenGC, including a higher number of WHO grade 2 meningiomas, with male preponderance, *NF2*-deficiency, a higher frequency of necrosis, a higher proliferation index, chromosomal instability with 1p loss, and shorter recurrence-free survival [42].

## 7. Conclusions

The 2021 WHO Classification includes molecular features, that is, *pTERT* mutation and *CDKN2A/B* HoDe, in meningioma grading for the first time, thus allowing the identification of some meningiomas devoid of malignant histological features but prone to a higher recurrence risk. However, owing to the rarity of these genetic alterations in recurring meningiomas, using this approach still leaves the issue of predicting the recurrence risk of most grade 2 meningiomas unsolved. In recent years, several sophisticated studies have shown that meningiomas can be classified using the integration of DNA methylation profiling, transcriptomics, and gene sequencing into three main groups, which can complement WHO grading for the prediction of clinical outcomes. Meningiomas in the first group are devoid of *NF2* alterations and chromosomal instability; may feature *AKT1*, *TRAF7*, or *KLF4* mutations; have the best prognosis; and are expected to respond to cytotoxic drugs. Meningiomas in the second group feature *NF2* inactivation, are devoid of chromosomal instability, are enriched in immune cells, and have an intermediate prognosis. Finally, meningiomas in the third group have a high chromosomal instability and proliferation index, may feature *pTERT* mutations and/or *CDKN2A/B* HoDe, have the worst prognosis, and are resistant to cytotoxic drugs. Although this classification may not be easily applied in clinical practice, proteomic studies have suggested that different groups can be recognized using specific immunostaining. 

In conclusion, the knowledge of the meningioma molecular landscape has greatly expanded over the last few years. Although this information has not been included in the current WHO 2021 Classification, a revolution in the grading scheme for meningiomas is expected over the next few years.

## Figures and Tables

**Table 1:** Criteria for meningioma grading according to the WHO 2016 and WHO 2021 Classifications.

Meningioma Grading According to WHO		
	WHO 2016	WHO 2021 *
Grade	Criteria	
**1**	**Histological**	
	Histotypes: Meningothelial Fibrous Transitional Psammomatous Angiomatous Microcystic Secretory Lymphoplasmacyte-rich Metaplastic	Lack of criteria consistent with grade 2 and 3
**2**	**Histological**	
	Chordoid	
	Clear cell	
	Atypical histotype:	All other subtypes:
4–19 mitotic figures/10 HPF and/or brain invasion and/or 3 minor criteria:		
	(1) Increased cellularity (2) Small cell with high N/C ratio (3) Macronucleoli (4) Patternless (sheet-like) growth (5) Foci of ‘spontaneous’ or geographic necrosis	
**3**	**Histological**	**Molecular**
	Papillary	*TERT* promoter mutation
	Rhabdoid	and/or *CDKN2A/B* homozygous deletion
	Anaplastic:	All subtypes:
	≥20 mitotic figures/10 HPF and/or frank anaplasia (sarcoma- carcinoma- or melanoma-like morphology	

* Grading applies to all subtypes, with the exception of chordoid and clear cells, which are considered grade 2 independent of their histopathological features. N/C: nuclear/cytoplasmic. HPF: high-power fields.

**Table 2:** Different meningioma classification system based on DNA methylation profiling.

Methylation-Based Classification of Meningiomas					
Authors	Technique	Group (Subgroup)	WHO Grade	Genetics	Recurrence Risk
Kishida et al. [27]	levels of methylation of 5 arbitrarily selected genes (*REC8*, *CHAD*, *HIF3A*, *UPK3A* and *SPOCK2*)	Hypomethylated			Low
		Hypermethylated			High
Olar et al. [28]	Classification based on the methylation of 283 CpG loci	MM-FAV (hypomethylated)	1, 2	Low CNA	Low
		MM-UNFAV (hypermethylated)	1, 2, 3	High CNA	High
Sahm et al. [29]	Genome-wide methylation analysis	A (MC ben-1)	Mostly 1	*NF2* mut 22q loss	Low
		A (MC ben-2)	Mostly 1	*NF* mut 22q loss	Low
		A (MC ben-3)	Mostly 1	*TRAF7*, *AKT1*, *KLF4* mut	Low
		A (MC int-A)	Mostly 1, 2	*NF2* mut 22q, 1p loss	Intermediate
				*NF2*, *pTERT* mut	
		B (MC int-B)	Mostly 2	*CDKN2A/B* HD	Intermediate
				1p, 22q loss	
		B (MC int-B)	Mostly 3	*NF2*, *pTERT* mut *CDKN2A/B* HD 1p, 22q, 10 loss	High
Katz et al. [30]	H3 K27me3 immunostaining	H3 K27me3 retained	1, 2, 3	More frequent *NF2* mut	Low
		H3K27me3 lost	1 (2%) 2 (11%) 3 (21%)		High

CNA: copy number alterations. Mut: mutation.

**Table 3:** Different proposed molecular classifications of meningiomas, resulting from the integration of methylome profiling, transcriptome, proteome, and genetic analyses, and achieving overlapping results.

Molecular Classification of Meningiomas				
Patel et al. [38]	Type A	Type B	Type C	
Genetic alterations	Mutations in *TRAF7*, *KLF4*, and *AKT1*	*NF2* mutations; Loss of chr22q	*NF2* mutation; 1p and 22q losses	
Outcome	Longer RFS	Longer RFS	Shorter RFS	
Proliferation index	Low	Intermediate	High	
Proteomic features		Loss of PCR2 complex function	Loss of DREAM complex function	
**Nassiri et al.** [39]	**MG2 (NF2 wildtype)**	**MG1 (immunogenic)**	**MG3 (hypermetabolic)**	**MG4 (proliferative)**
Genetic alterations	Mutations in *TRAF7*, *KLF4*, and *AKT1* or chromosome 5 polysomy	*NF2* mutations; loss of chr22q	*NF2* mutation; chromosomal losses	
Outcome	Intermediate RFS	Longest RFS	Shortest RFS	
Preteomic features	SCGN	S100A	ACADL	MCM2
**Choudhury et al.** [40]	**NF2/merlin intact**	**Immune enriched**	**Hypermitotic**	
Genetic alterations	*NF2* wild type	*NF2* mutations; Loss of chr22q	Multiple chromosomal losses; *CDKN2A/B* HoDe; *pTERT* mutation	
Outcome	Best prognosis	Intermediate prognosis	Poor prognosis	
Other features	Responsive to cytotoxic treatment	Enrichment in immune cells and lymphatics	Resistance to cytotoxic therapy	

RFS: recurrence-free survival. HoDe: homozygous deletion.
